# Supplementary material for: A protocol for the economic evaluation of the smoking Cessation in Pregnancy Incentives Trial III (CPIT III)
Source: BMJ Open. 2020 Oct 27;10(10):e038827. doi: 10.1136/bmjopen-2020-038827 (PMC7592273; doi:10.1136/bmjopen-2020-038827)
Supplement: Author's manuscript [file bmjopen-2020-038827.draft_revisions.pdf]

BMJ Open is committed to open peer review. As part of this commitment we make the peer review history of every article we publish publicly available.

When an article is published we post the peer reviewers' comments and the authors' responses online. We also post the versions of the paper that were used during peer review. These are the versions that the peer review comments apply to.

The versions of the paper that follow are the versions that were submitted during the peer review process. They are not the versions of record or the final published versions. They should not be cited or distributed as the published version of this manuscript.

BMJ Open is an open access journal and the full, final, typeset and author-corrected version of record of the manuscript is available on our site with no access controls, subscription charges or pay-per-view fees (http://bmjopen.bmj.com).

If you have any questions on BMJ Open's open peer review process please email info.bmjopen@bmj.com

A protocol for the economic evaluation of the Smoking Cessation in Pregnancy Incentives Trial (CPIT III)

Journal:	*BMJ Open*
Manuscript ID	bmjopen-2020-038827
Article Type:	Protocol
Date Submitted by the Author:	26-Mar-2020

Complete List of Authors:	McMeekin, Nicola; University of Glasgow Institute of Health and Wellbeing, HEHTA<br>Sinclair, Lesley; The University of Edinburgh Usher Institute of Population Health Sciences and Informatics<br>Bauld, Linda; University of Edinburgh, Usher Institute of Population Health Sciences and Informatics<br>Tappin, David; University of Glasgow, Scottish Cot Death Trust, West Glasgow Ambulatory Care Hospital, Glasgow G3 8SJ<br>Mitchell, Alex; University of York, York Trials Unit, Department of Health Sciences<br>Boyd, Kathleen; University of Glasgow, Health Economics & Health Technology Assessment

Keywords:	HEALTH ECONOMICS, PUBLIC HEALTH, Maternal medicine < OBSTETRICS

SCHOLARONE™ Manuscripts

I, the Submitting Author has the right to grant and does grant on behalf of all authors of the Work (as defined in the below author licence), an exclusive licence and/or a non-exclusive licence for contributions from authors who are: i) UK Crown employees; ii) where BMJ has agreed a CC-BY licence shall apply, and/or iii) in accordance with the terms applicable for US Federal Government officers or employees acting as part of their official duties; on a worldwide, perpetual, irrevocable, royalty-free basis to BMJ Publishing Group Ltd ("BMJ") its licensees and where the relevant Journal is co-owned by BMJ to the co-owners of the Journal, to publish the Work in this journal and any other BMJ products and to exploit all rights, as set out in our licence.

The Submitting Author accepts and understands that any supply made under these terms is made by BMJ to the Submitting Author unless you are acting as an employee on behalf of your employer or a postgraduate student of an affiliated institution which is paying any applicable article publishing charge ("APC") for Open Access articles. Where the Submitting Author wishes to make the Work available on an Open Access basis (and intends to pay the relevant APC), the terms of reuse of such Open Access shall be governed by a Creative Commons licence – details of these licences and which Creative Commons licence will apply to this Work are set out in our licence referred to above.

Other than as permitted in any relevant BMJ Author's Self Archiving Policies, I confirm this Work has not been accepted for publication elsewhere, is not being considered for publication elsewhere and does not duplicate material already published. I confirm all authors consent to publication of this Work and authorise the granting of this licence.

For peer review only

Title:

A protocol for the economic evaluation of the Smoking Cessation in Pregnancy Incentives Trial (CPIT III)

Authors: McMeekin N., Sinclair, L., Bauld, L., Tappin, D., Mitchell, A., Boyd K.A

Nicola Mcmeekin[1] nicola.mcmeekin@glasgow.ac.uk
Lesley Sinclair[2] lesley.sinclair@ed.ac.uk
Linda Bauld[2] linda.bauld@ed.ac.uk
David M Tappin[3] david.tappin@glasgow.ac.uk
Alex Mitchell[4] alex.mitchell@york.ac.uk
Kathleen Boyd[1] kathleen.boyd@glasgow.ac.uk

[1] Health Economics & Health Technology Assessment, Institute of Health & Wellbeing, 1 Lilybank Gardens, University of Glasgow, Glasgow, G12 8RZ, UK

[2] Usher Institute, University of Edinburgh, Teviot Place, Edinburgh, EH8 9AG, UK

[3] Scottish Cot Death Trust, West Glasgow Ambulatory Care Hospital, Glasgow G3 8SJ, UK

[4] York Trial Unit, Department of Health Sciences, Faculty of Science, University of York, Alcuin Research Resource Centre, Heslington, York, YO10 5DD, UK

Corresponding author: Nicola McMeekin, Health Economics & Health Technology Assessment, Institute of Health & Wellbeing, 1 Lilybank Gardens, University of Glasgow, Glasgow, G12 8RZ, UK. nicola.mcmeekin@glasgow.ac.uk 0141 330 5010

Acknowledgements to the wider CPIT team: Margaret McFadden, Helen Tilbrook, Ada Keding, Judith Watson, Frank Kee, David Torgerson, Catherine Hewitt, Jennifer McKell, Pat Hoddinott, Fiona M Harris, Isabelle Uny, Michael Ussher

Keywords: smoking cessation; financial incentives; economic evaluation; cost-effectiveness analysis; pregnancy

For peer review only - http://bmjopen.bmj.com/site/about/guidelines.xhtml

ABSTRACT

Introduction

Smoking results in an average 10-year loss of life, but smokers who permanently quit before age 40 can expect a near normal lifespan. Pregnancy poses a good opportunity to help women to stop; around 80% of women in the UK have a baby, most less than age 40. Smoking prevalence during pregnancy is high; 17-23% in the UK. Smoking during pregnancy causes low birth weight and increases the risk of premature birth. Post-birth, passive smoking is linked to sudden infant death syndrome, respiratory diseases and increased likelihood of taking up smoking. These risks impact the long-term health of the child with associated increase in health costs. Emerging evidence suggests that offering financial incentives to pregnant women to quit is highly cost-effective.

This protocol describes the economic evaluation of a multi-centre randomised controlled trial designed to establish whether offering financial incentives, in addition to usual care, is effective and cost-effective in helping pregnant women to quit.

Methods and Analysis

The economic evaluation will identify, measure and value resource use and outcomes from the CPIT III trial, comparing participants randomised to either usual care or usual care plus up to £400 financial incentives. Within-trial and long-term analyses will be conducted from a National Health Service and Personal Social Services perspective, the outcome for both analyses will be quality adjusted life-years measured using EQ-5D-5L. Patient level data collected during the trial will be used for the within-trial analysis, with an additional outcome of cotinine validated quit rates at 34-38 weeks gestation and six-months post-partum. The long-term model will be informed by data from the trial and published literature.

Ethics and Dissemination

Ethics approval received from NHS West of Scotland Research Ethics Committee-2, August 2017. Dissemination through peer-reviewed publications and presentations.

Registration details

Trial registration number: ISRCTN15236311, date registered 09/10/2017
https://doi.org/10.1186/ISRCTN15236311

Strengths and limitations of this study:

- Prospectively designed economic evaluation of a phase III randomised controlled trial with sites across the UK
- Preference-based (utility) outcome measures at late-stage pregnancy and 6-months post-partum to enable decision-making
- 6-months post-partum follow-up is the longest we are aware of, making this a novel study

For peer review only - http://bmjopen.bmj.com/site/about/guidelines.xhtml

- Lifetime extrapolation includes costs and outcomes for both mother and infant
- Challenges relate to different smoking cessation service delivery at each trial site

INTRODUCTION

Problem

Smoking is the principal preventable cause of cancer, with 64,000 new cases annually in the UK(1). Levels of smoking reflect levels of inequality; in 2016 in England 7.9% of adults smoked in the least deprived areas compared to 27.2% in the most deprived areas(2). Lifelong smokers lose 10 years of life, however, smokers who quit before 40 years of age can expect a near to normal lifespan (3). Levels of smoking during pregnancy are high; in 2006 in England 17% of pregnant women smoked, in Scotland in 2005 this figure was 23%. Smoking during pregnancy is a recognised cause of low birth weight and increases the risk of premature birth by 27%. In addition to complications during pregnancy, after birth the consequences of passive smoking on the child are substantial: passive smoking is linked to sudden infant death syndrome, lower respiratory diseases, asthma and impaired lung function. Research shows that children in a house where parents or siblings smoke are 90% more likely to smoke themselves (4). Around 80% of UK women have a baby(5), most of whom are less than 40 years of age. Pregnancy is a good opportunity to help women quit smoking, and also decreases the likelihood of the baby becoming a smoker in later life(4). Helping pregnant women to quit smoking can potentially help tackle inequalities and lift families out of poverty(6). However, few of about 130,000 UK pregnant smokers quit. In the UK all pregnant women are offered NHS smoking cessation services, with free Nicotine Replacement Therapy (NRT), however, only 10% use these services and as few as 3% quit(4).

Evidence

Financial incentives are becoming a popular approach to changing behaviour that will lead to healthier lifestyles (7). A recent Cochrane review which identified 33 trials (over 21,600 participants) found that nine trials following 2,273 pregnant smokers confirmed the efficacy of financial incentives in this group (Notley). A single site study (CPIT II) found offering financial incentives helped pregnant women in Glasgow, Scotland to quit(8). The CPIT II study estimated a cost per quality adjusted life-year of less than £500, which is considered to be highly cost-effective(9).

CPIT III

CPIT III builds on the evidence from CPIT II (8) by including further UK sites and a six-month post-partum follow-up. It is a multi-centre randomised controlled trial which

For peer review only - http://bmjopen.bmj.com/site/about/guidelines.xhtml

aims to assess the effectiveness and cost-effectiveness of adding financial incentives to usual care to increase the smoking cessation rate among pregnant women. The primary outcome is smoking at 34-38 weeks gestation, self-reported, with those reporting as quit undertaking urine or saliva confirmatory biochemical testing. The secondary outcomes comprise: engaging with smoking cessation services and setting a quit date; smoking cessation at four weeks post quit date and six months post-partum (with those reporting as quit confirmed by biochemical testing); continuous abstinence from later pregnancy to six months post-partum; birth weight; cost-effectiveness and process evaluation.

Participants will be pregnant women who self-report as smokers, and inclusion criteria are women aged 16 years or older, less than 24 weeks pregnant and English speaking. The target recruitment number is 940, to give 90% power and 5% significance to show a doubling of quit rate from 7% in the control arm to 14% with financial incentives, allowing 15% loss to follow-up. The estimate of 7% in the control arm was derived from a pilot trial in Glasgow (8.6% abstinent) (8) and in two other recent large trials of pregnancy cessation interventions, conducted in regions where CPIT III will recruit; abstinent 6.4% (10) and 7.6%(11). The average control group abstinence rate was 7.5%. The estimate of 14% in the intervention arm is based on both the cessation rate in CPIT II (22.5%) plus a reflection of what is considered clinically important. A gain of 7% to 14% would be considered clinically important and is comparable to pharmaceutical aids in non-pregnant smokers (12).

Participants will be recruited from seven sites in England, Scotland and Northern Ireland, identified at routine maternity booking appointments. They will be randomised to either receive financial incentives plus usual smoking cessation support or usual smoking cessation support only. The incentives are presented in Table 1.

Both groups will be offered local smoking cessation support and a research participation voucher. This shopping voucher for £50 is issued if data is provided for the primary outcome in late pregnancy and a further £25 shopping voucher issued if data is provided for the secondary outcome at six months post-partum. Participants can still receive the £25 shopping voucher if they provide data for the secondary outcome but do not provide data for the primary outcome.

Participants in the intervention arm will also receive up to £400 in shopping vouchers; £50 for attending an initial appointment with local smoking cessation services and setting a quit date, £50 if verified quit at 4 weeks post quit date, £100 if verified quit at 12 weeks post quit date and £200 if verified quit at 34-38 weeks gestation (primary outcome point). All incentive payments, apart from the first, require quit to be verified by carbon monoxide (CO) breath test. Cotinine/anabasine will also be used to confirm non-smoking status at the primary outcome and six months post-partum. Participants in the incentives group not attending smoking cessation services can still receive the final £200 shopping voucher if they are CO verified quit at the primary endpoint of 34-38 weeks gestation.

For peer review only

Further details on the CPIT III trial are detailed in the main study protocol paper which is available elsewhere (13).

Table 1: Intervention and research participation incentives

Time point	Intervention	Control
Initial local smoking cessation services meeting and setting quit date	£50 voucher	N/A
Verified quit at four weeks post quit date	£50 voucher	N/A
Verified quit at 12 weeks post quit date	£100 voucher	N/A
Verified quit at 34-38 weeks gestation	£200 voucher	N/A
Providing data for primary outcome	£50 voucher for research participation	£50 voucher for research participation
Providing data for secondary outcome (quit six-month post-partum)	£25 voucher for research participation	£25 voucher for research participation

This paper describes the protocol for the economic evaluation of the CPIT III trial. The aim of the economic evaluation is to explore the cost-effectiveness of including financial incentives alongside usual care to increase the quit rate of pregnant women who smoke.

METHODS AND ANALYSIS

Overview

The economic evaluation will be conducted from the perspective of the UK National Health Service (NHS) and Personal Social Services (PSS), for price year 2020. Two analyses will be carried out; a within trial analysis and a long-term lifetime analysis. The within trial results will be presented as an incremental cost per quitter and an incremental cost per quality adjusted life-year (QALY) gained. The lifetime model results will be reported as incremental cost per quality adjusted life-year gained. For both analyses results will be assessed against the current NICE threshold of £20,000 to £30,000(14) per QALY gained. The economic evaluation will adhere to best practice and guidelines (15, 16).

An overview of this analysis is illustrated in **Error! Reference source not found.** below; the participants (pregnant smokers) are randomised to either the intervention or control arm. The primary outcome of the trial is quit at 34–38 weeks gestation and both this trial primary outcome and the economic outcome of QALYs will be used in the cost-effectiveness analysis.

For peer review only - http://bmjopen.bmj.com/site/about/guidelines.xhtml

Figure 1: Decision tree including intervention and outcomes

The analysis will be conducted using the statistical analysis package STATA version 16 (StataCorp. 2019. Stata Statistical Software: Release 16. College Station, TX: StataCorp LLC).

Within-trial analysis

Patient level data on resource use and outcomes will be routinely collected during the trial and used in the economic evaluation. The time horizon for the within-trial analysis is that of the trial; from time of maternity booking (less than 24 weeks gestation) to late-pregnancy (34-38 weeks gestation) and six months post-partum. No discounting of costs and outcomes is required as this time horizon is less than one year. The analysis will be undertaken based on an intention to treat (ITT) approach.

Resource use

There are four main resource use groups of relevance informing the within trial analysis: smoking cessation support (professional time), intervention costs (financial incentives), NRT and neonatal care.

Service delivery of smoking cessation support and prescribing of NRT is likely to vary between trial sites. Health economists will work with each site to establish service delivery and set up and maintain procedures for prospective resource use data. To date methods used for collecting resources include routinely collected NHS data and bespoke Excel spreadsheets.

The trial data management system will record when shopping vouchers are sent to participants and a standard postal cost will be assigned to each voucher posted. Vouchers are sent at first consultation, four and 12 weeks post-quit, late pregnancy (34-38 weeks) and six months post-partum.

The child's birthweight and date of delivery will also be collected enabling pre-term status to be derived. Assumptions will be made about the levels of neo-natal care required based on this birthweight and prematurity/pre-term status.

Unit Costs

Unit costs will be obtained from routine sources:

- British National Formulary – NRT (17)
- Personal Social Service Research Unit – Smoking cessation advisor/pharmacist(18)
- Trial data management system – shopping vouchers/postage
- NHS health resource use – NHS reference costs, ISD and literature.

For peer review only - http://bmjopen.bmj.com/site/about/guidelines.xhtml

Costs will be for the price year 2020, expressed in pounds sterling (GBP £) and inflated if necessary using the Health Services (HS) index(19). For publication in journals, final costs may also be presented in US dollars, converted using the exchange rate, so that the results will be appropriate for an international audience.

Unit cost information will be combined with the resource use data collected in the trial to estimate the total cost per participant in each trial arm. These participant total costs will be aggregated to estimate the total cost of each trial arm and subsequently the average cost per participant for each intervention.

Regression analysis will be conducted to explore the effect that baseline variables have on the cost of each intervention, such as EQ-5D-5L score, site, gestational age at booking. Variables included will be in line with the statistical analysis plan and main analysis.

Outcomes

There will be two economic outcomes: the within trial quit rate and QALY.

The primary outcome of the trial is smoking status at 34-38 weeks gestation. This indicator will inform the incremental cost per quitter analysis. As this is an intermediate outcome and not a measure of health we are also conducting a within trial incremental cost per QALY analysis

The QALY is a combination of quality of life and quantity of life. Quality of life will be measured using the EQ-5D-5L questionnaire (12), which measures five dimensions: mobility, self-care, usual activities, pain/discomfort and anxiety/depression, with five levels: no problems, slight problems, moderate problems, severe problems and extreme problems. The EQ-5D-5L will be completed by participants at three time points: baseline, late pregnancy (34-38 weeks gestation) and six months post-partum.

The EQ-5D-5L is converted to utilities using value sets giving a utility value of between -0.59 and 1. A utility value of 1 represents full health, 0 represents death and between 0 and -0.59 represents a state worse than death. We will map the EQ-5D-5L results to the EQ-5D-3L UK value set (ref) in accordance to the recommendation in the current position statement from NICE. (13, 14)
The quantity of life element of the QALY will be the length of time the participant remains in the trial, using standard QALY calculation applying an area under the curve approach(20). For example, for a participant recruited at 20 weeks gestation who has primary outcome data collection at 38 weeks the calculation for a quality adjusted life-year would be as follows:

$$(\text{Utility at 38 weeks gestation} - \text{Utility at 20 weeks gestation}) \times \frac{38-20}{52}$$

For peer review only - http://bmjopen.bmj.com/site/about/guidelines.xhtml

When combining the utility score generated from the EQ-5D-5L with quantity of life to give a QALY, standard area-under-the-curve methods(21) will be employed; weighting utilities by the duration of each time interval. Any change in utility between time points will be treated as linear.

A regression analysis will be carried out in line with the main analysis and statistical analysis plan exploring the effect that baseline variables have on the outcomes, such as site, gestational age at booking and in particular baseline utilities(16).

Analysis of costs and effects

Missing health economic data will be dealt with in line with best practice guidance(17): a descriptive analysis of the missing data will be carried out and assumptions will be made to ascertain the type of missingness, following this an appropriate method for handling missing data will be chosen, and finally, sensitivity analysis will be carried out around assumptions as appropriate.

Missing outcome data will be dealt with using the same methods used in the main trial analysis in consultation with the trial statistician.

The cost-effectiveness analysis will bring together the estimates of cost and effect as described above, by estimating an incremental cost effectiveness ratio (ICER) of cost per quitter and per QALY. The difference between mean costs will be divided by the difference in mean QALYs and quit rates to calculate the ICER, the formula for this is presented below. The control arm is denoted by ‘A’ and intervention (financial incentives) arm by ‘B’.

$$\text{ICER} = \frac{C_B - C_A}{E_B - E_A}$$

Costs, QALYs and quit rates per participant per trial arm will be presented as means and standard deviations. Differences in means between trial arms will be presented with 95% confidence intervals (CI) using generalised linear models with appropriate link and family functions.

Uncertainty

The effect of uncertainty on the results will be explored using non-parametric bootstrapping techniques and the resulting samples will be plotted on a cost-effectiveness plane in order to graphically represent uncertainty (22). Cost-effectiveness acceptability curves (CEACs) will then be used to present uncertainty in terms of willingness to pay under various monetary thresholds (23). 95% confidence intervals for the ICER will be presented using the results of the bootstrapping. If appropriate, 95% confidence intervals for the ICER will be estimated using Fieller’s Theorem, a technique that includes any correlation between cost and outcome(24).

For peer review only - http://bmjopen.bmj.com/site/about/guidelines.xhtml

Any sub-group analyses deemed appropriate in the statistical analysis plan will also be considered for the economic analysis. This will include an analysis using self-reported smoker/quitter extracted from case notes where the trial team have not been able to establish smoking status via direct contact with participants.

Two sensitivity analyses will be run; using miscarriage data from the trial and exploring the effects of gaming (where the participant self-reports as a quitter but is still smoking) in the trial.

Long-term model

The time horizon for the long-term economic model is lifetime. Costs and effects will be discounted at 3.5% annually in line with recommendations from NICE(16). The within trial analysis only considers the costs and effects of the pregnant woman and neonatal costs. The long-term model will also include the costs and effects related to the child. The rationale for this is that children of smokers are more likely to smoke themselves and will also be exposed to second-hand smoke, increasing the likelihood of smoking related diseases(4).

Model structure

The analysis will utilise and adapt a published probabilistic decision analytic Markov model, the Economics of Smoking in Pregnancy (ESIP) model (25). The model will comprise two sections; one to capture the lifetime costs and outcomes for the mother, and one to capture the lifetime costs and outcomes of the child(21). The ESIP model was the first model specifically developed to include future potential cost savings and improvement in health for both mother and child. A short-term cost-effectiveness analysis can be misleading as the benefits of stopping smoking are not captured in a short time horizon, which is why it is important to incorporate future benefits to assess the full impact of offering financial incentives to pregnant smokers.
The mother's portion of the model will include: the costs of smoking cessation services, financial incentives, NRT use and smoking related diseases, plus data on relapse rates and transition to smoking related diseases if the woman relapses. The child's part of the model will include: costs associated at birth for children with smoking mothers if appropriate (premature birth and underweight new-borns), outcomes related to exposure to second-hand smoke in the home (ie asthma and longer term smoking related diseases), smoking uptake rates for children of smokers and associated smoking related diseases.
The trial quit rate at six months post birth will be used to predict smoking relapse post-partum. The final utility estimates for patient level data from the post-partum follow-up will also be used as a point for extrapolation for lifetime analysis (rather than assuming background population utility values).

Figure 2 depicts the likely structure of the long-term model(26). The model is split between mother and infant; the first 'Within pregnancy' section on the left illustrates within pregnancy outcomes and related costs, the middle 'Childhood' section depicts the infant's childhood from birth to 15 years old and the last section 'Lifetime' on the right, depicts lifetime for both mother and infant.

For peer review only - http://bmjopen.bmj.com/site/about/guidelines.xhtml

Resource use and outcomes collected during the trial will be extrapolated and supplemented with published costs from the literature.

Figure 2: Model structure (Jones et al) (26)

Analysis

The lifetime analysis results will be presented as cost per QALY gained for both the mother and child, separately and combined.

A probabilistic sensitivity analysis will be included and appropriate sensitivity analysis will explore underlying assumptions used in the model, including:

- Re-analysis to account for gaming identified
- Self-reported outcomes

And potentially:

- Varying amount of incentive
- Applying alternative discount rates
- Varying relapse rates

Patient and Public Involvement

No patient involved.

Ethics and dissemination

Ethics approval was received from NHS West of Scotland Research Ethics Committee 15th August 2017. Results of the main trial and economic evaluation will be disseminated through peer-reviewed publications and presentations.

References

1. Parkin DM, Boyd L, Walker LC. The fraction of cancer attributable to lifestyle and environmental factors in the UK in 2010 Summary and conclusions. British Journal of Cancer. 2011;105:S77-S81.

2. Office of National Statistics. Likelihood of smoking four times higher in Englandâ€™s most deprived areas than least deprived - Office for National Statistics 2019 [Available from: https://www.ons.gov.uk/peoplepopulationandcommunity/healthandsocialcare/drugusealcoholandsmoking/articles/likelihoodofsmokingfourtimeshigherinenglandsmostdeprivedareasthanleastdeprived/2018-03-14.

3. Doll R, Peto R, Boreham J, Sutherland I. Mortality in relation to smoking: 50 years' observations on male British doctors. Bmj-British Medical Journal. 2004;328(7455):1519-28.

4. Leonardi-Bee J, Jere ML, Britton J. Exposure to parental and sibling smoking and the risk of smoking uptake in childhood and adolescence: a systematic review and meta-analysis. Thorax. 2011;66(10):847-55.

5. Portani M, Whitworth S. A comparison of the characteristics of childless women and mother in the ONS longitudinal study. Population trends. 2009(136):10-20.

For peer review only - http://bmjopen.bmj.com/site/about/guidelines.xhtml

6. Belvin C, Britton J, Holmes J, Langley T. Parental smoking and child poverty in the UK: an analysis of national survey data. Bmc Public Health. 2015;15.
7. Vlaev I, King D, Darzi A, Dolan P. Changing health behaviors using financial incentives: a review from behavioral economics. Bmc Public Health. 2019;19(1).
8. Tappin D, Bauld L, Purves D, Boyd K, Sinclair L, MacAskill S, et al. Financial incentives for smoking cessation in pregnancy: randomised controlled trial. BMJ-British Medical Journal. 2015;350.
9. Boyd KA, Briggs AH, Bauld L, Sinclair L, Tappin D. Are financial incentives cost-effective to support smoking cessation during pregnancy? Addiction. 2016;111(2):360-70.
10. Ussher M, Lewis S, Aveyard P, Manyonda I, West R, Lewis B, et al. Physical activity for smoking cessation in pregnancy: randomised controlled trial. Bmj-British Medical Journal. 2015;350.
11. Coleman T, Cooper S, Thornton JG, Grainge MJ, Watts K, Britton J, et al. A Randomized Trial of Nicotine-Replacement Therapy Patches in Pregnancy. New England Journal of Medicine. 2012;366(9):808-18.
12. Cahill K, Stevens S, Perera R, Lancaster T. Pharmacological interventions for smoking cessation: an overview and network meta-analysis. The Cochrane database of systematic reviews. 2013(5):CD009329-CD.
13. Sinclair L, McFadden M, Tilbrook H, Mitchell A, Keding A, Watson J, et al. The smoking cessation in pregnancy incentives trial (CPIT): study protocol for a phase III randomised controlled trial. Trials. 2020;21(1).
14. National Institute for Health and Care Excellence. Interim methods guide for developing service guidance 2014: NICE; 2016 [Available from: https://www.nice.org.uk/process/pmg8/chapter/5-developing-a-conceptual-model.
15. Husereau D, Drummond M, Petrou S, Carswell C, Moher D, Greenberg D, et al. Consolidated Health Economic Evaluation Reporting Standards (CHEERS) statement. European Journal of Health Economics. 2013;14(3):367-72.
16. Excellence NIfHaC. Guide to the methods of technology appraisal 2013 | foreword | Guidance and guidelines | NICE. 2015.
17. Joint Formulary Committee. British National Formulary 2018 [Available from: https://www.medicinescomplete.com/#/.
18. Curtis L, Burns A. Unit Costs of Health and Social Care 2017 | PSSRU 2018 [Available from: https://www.pssru.ac.uk/project-pages/unit-costs/unit-costs-2017/.
19. Curtis L. Unit Costs of Health and Social Care 2018 | PSSRU 2019 [Available from: https://www.pssru.ac.uk/project-pages/unit-costs/unit-costs-2018/.
20. Manca A, Hawkins N, Sculpher MJ. Estimating mean QALYs in trial-based cost-effectiveness analysis: the importance of controlling for baseline utility. Health Economics. 2005;14(5):487-96.
21. Billingham LJ, Abrams KR, Jones DR. Methods for the analysis of quality-of-life and survival data in health technology assessment. Health technology assessment (Winchester, England). 1999;3(10):1-152.
22. Briggs A, Claxton K, Sculpher M. Decision Modelling for Health Economic Evaluation. 2011 ed. Gray A, Briggs A, editors. New York: Oxford University Press; 2006.
23. Fenwick E, Claxton K, Sculpher M. Representing uncertainty: The role of cost-effectiveness acceptability curves. Health Economics. 2001;10(8):779-87.
24. Fieller EC. SOME PROBLEMS IN INTERVAL ESTIMATION. Journal of the Royal Statistical Society Series B-Statistical Methodology. 1954;16(2):175-85.
25. Jones M, Smith M, Lewis S, Parrott S, Coleman T. A dynamic, modifiable model for estimating cost-effectiveness of smoking cessation interventions in pregnancy: application to an RCT of self-help delivered by text message. Addiction. 2019;114(2):353-65.
26. Jones Mea. Economics of Smoking in Pregnancy (ESIP) Model - The University of Nottingham 2019 [Available from:

For peer review only - http://bmjopen.bmj.com/site/about/guidelines.xhtml

https://www.nottingham.ac.uk/research/groups/tobaccoandalcohol/smoking-in-pregnancy/esip/index.aspx.

Trial registration

Current Controlled Trials ISRCTN15236311 date registered 09/10/2017
https://doi.org/10.1186/ISRCTN15236311

Authors' contributions

NM and KAB designed the economic evaluation protocol and wrote the paper, LS, LB, DT and AM contributed to revising the content of the paper and contributed to editing.

Source of funding

Cancer research UK, Chief Scientist Office Scottish Government, Health and Social Care Services Northern Ireland, Chest Heart & Stroke Northern Ireland, Lullaby Trust, Scottish Cot Death Trust.

Competing interests statement

No authors have competing interests.

For peer review only

For peer review only - http://bmjopen.bmj.com/site/about/guidelines.xhtml

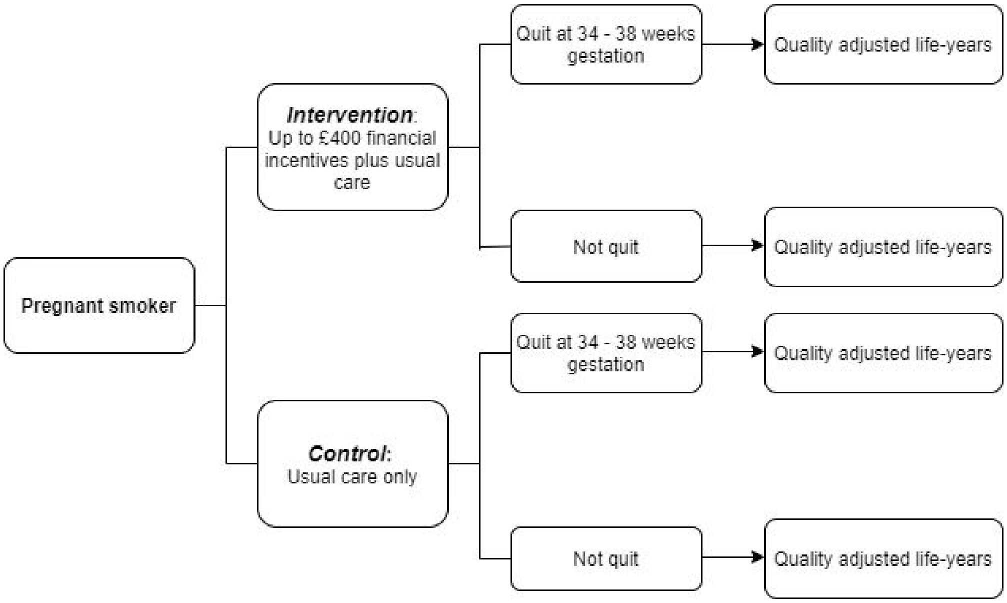

Figure 1: Decision including intervention and outcomes

222x132mm (72 x 72 DPI)

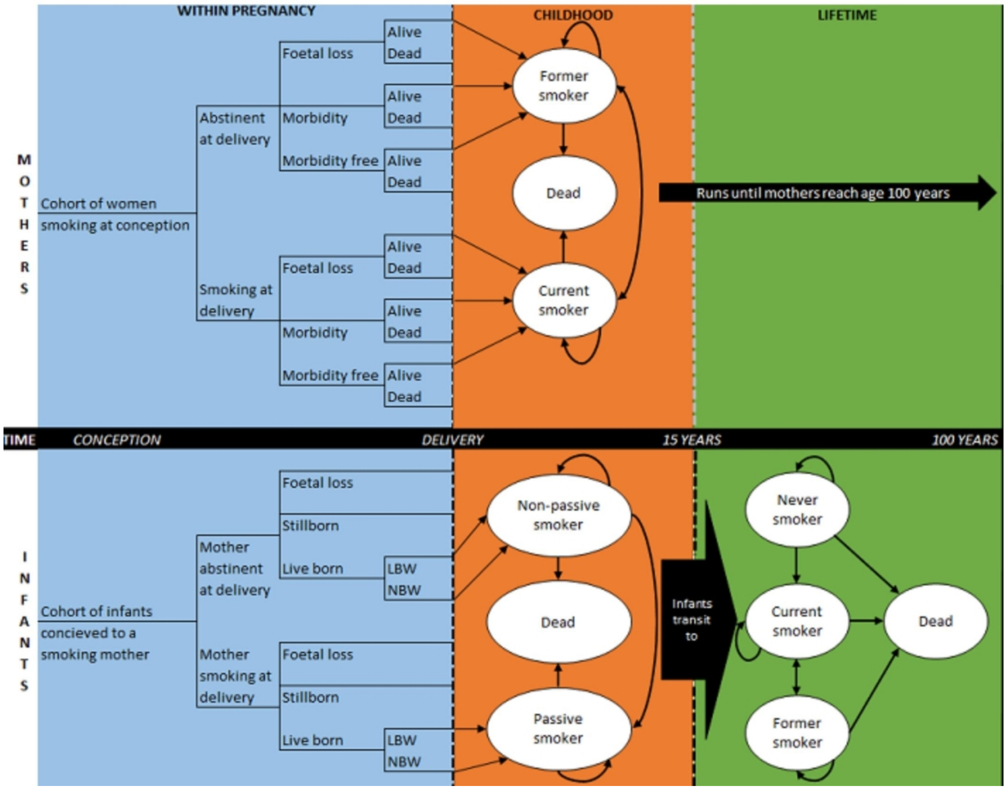

Figure 2: Model structure (Jones et al) (26)

562x442mm (96 x 96 DPI)

BMJ Open

A protocol for the economic evaluation of the Smoking Cessation in Pregnancy Incentives Trial (CPIT III)

Journal:	*BMJ Open*
Manuscript ID	bmjopen-2020-038827.R1
Article Type:	Protocol
Date Submitted by the Author:	03-Aug-2020
Complete List of Authors:	McMeekin, Nicola; University of Glasgow Institute of Health and Wellbeing, HEHTA<br>Sinclair, Lesley; The University of Edinburgh Usher Institute of Population Health Sciences and Informatics<br>Bauld, Linda; University of Edinburgh, Usher Institute of Population Health Sciences and Informatics<br>Tappin, David; University of Glasgow, Scottish Cot Death Trust, West Glasgow Ambulatory Care Hospital, Glasgow G3 8SJ<br>Mitchell, Alex; University of York, York Trials Unit, Department of Health Sciences<br>Boyd, Kathleen; University of Glasgow, Health Economics & Health Technology Assessment
**Primary Subject Heading**:	Health economics
Secondary Subject Heading:	Smoking and tobacco, Addiction
Keywords:	HEALTH ECONOMICS, PUBLIC HEALTH, Maternal medicine < OBSTETRICS

SCHOLARONE™
Manuscripts

I, the Submitting Author has the right to grant and does grant on behalf of all authors of the Work (as defined in the below author licence), an exclusive licence and/or a non-exclusive licence for contributions from authors who are: i) UK Crown employees; ii) where BMJ has agreed a CC-BY licence shall apply, and/or iii) in accordance with the terms applicable for US Federal Government officers or employees acting as part of their official duties; on a worldwide, perpetual, irrevocable, royalty-free basis to BMJ Publishing Group Ltd ("BMJ") its licensees and where the relevant Journal is co-owned by BMJ to the co-owners of the Journal, to publish the Work in this journal and any other BMJ products and to exploit all rights, as set out in our licence.

The Submitting Author accepts and understands that any supply made under these terms is made by BMJ to the Submitting Author unless you are acting as an employee on behalf of your employer or a postgraduate student of an affiliated institution which is paying any applicable article publishing charge ("APC") for Open Access articles. Where the Submitting Author wishes to make the Work available on an Open Access basis (and intends to pay the relevant APC), the terms of reuse of such Open Access shall be governed by a Creative Commons licence – details of these licences and which Creative Commons licence will apply to this Work are set out in our licence referred to above.

Other than as permitted in any relevant BMJ Author's Self Archiving Policies, I confirm this Work has not been accepted for publication elsewhere, is not being considered for publication elsewhere and does not duplicate material already published. I confirm all authors consent to publication of this Work and authorise the granting of this licence.

For peer review only

Title:
A protocol for the economic evaluation of the Smoking Cessation in Pregnancy Incentives Trial (CPIT III)

Authors: McMeekin N., Sinclair, L., Bauld, L., Tappin, D., Mitchell, A., Boyd K.A

Nicola Mcmeekin[1]	nicola.mcmeekin@glasgow.ac.uk
Lesley Sinclair[2]	lesley.sinclair@ed.ac.uk
Linda Bauld[2]	linda.bauld@ed.ac.uk
David M Tappin[3]	david.tappin@glasgow.ac.uk
Alex Mitchell[4]	alex.mitchell@york.ac.uk
Kathleen Boyd[1]	kathleen.boyd@glasgow.ac.uk

[1] Health Economics & Health Technology Assessment, Institute of Health & Wellbeing, 1 Lilybank Gardens, University of Glasgow, Glasgow, G12 8RZ, UK

[2] Usher Institute, University of Edinburgh, Teviot Place, Edinburgh, EH8 9AG, UK

[3] Scottish Cot Death Trust, West Glasgow Ambulatory Care Hospital, Glasgow G3 8SJ, UK

[4] York Trial Unit, Department of Health Sciences, Faculty of Science, University of York, Alcuin Research Resource Centre, Heslington, York, YO10 5DD, UK

Corresponding author: Nicola McMeekin, Health Economics & Health Technology Assessment, Institute of Health & Wellbeing, 1 Lilybank Gardens, University of Glasgow, Glasgow, G12 8RZ, UK. nicola.mcmeekin@glasgow.ac.uk 0141 330 5010

Acknowledgements to the wider CPIT team: Margaret McFadden, Helen Tilbrook, Ada Keding, Judith Watson, Frank Kee, David Torgerson, Catherine Hewitt, Jennifer McKell, Pat Hoddinott, Fiona M Harris, Isabelle Uny, Michael Ussher

Keywords: smoking cessation; financial incentives; economic evaluation; cost-effectiveness analysis; pregnancy

For peer review only - http://bmjopen.bmj.com/site/about/guidelines.xhtml

ABSTRACT

Introduction

Smoking results in an average 10-year loss of life, but smokers who permanently quit before age 40 can expect a near normal lifespan. Pregnancy poses a good opportunity to help women to stop; around 80% of women in the UK have a baby, most less than age 40. Smoking prevalence during pregnancy is high; 17-23% in the UK. Smoking during pregnancy causes low birth weight and increases the risk of premature birth. Post-birth, passive smoking is linked to sudden infant death syndrome, respiratory diseases and increased likelihood of taking up smoking. These risks impact the long-term health of the child with associated increase in health costs. Emerging evidence suggests that offering financial incentives to pregnant women to quit is highly cost-effective.

This protocol describes the economic evaluation of a multi-centre randomised controlled trial (CPIT III) designed to establish whether offering financial incentives, in addition to usual care, is effective and cost-effective in helping pregnant women to quit.

Methods and Analysis

The economic evaluation will identify, measure and value resource use and outcomes from CPIT III, comparing participants randomised to either usual care or usual care plus up to £400 financial incentives. Within-trial and long-term analyses will be conducted from a National Health Service and Personal Social Services perspective, the outcome for both analyses will be quality adjusted life-years measured using EQ-5D-5L. Patient level data collected during the trial will be used for the within-trial analysis, with an additional outcome of cotinine validated quit rates at 34-38 weeks gestation and six-months post-partum. The long-term model will be informed by data from the trial and published literature.

Ethics and Dissemination

Ethics approval received from NHS West of Scotland Research Ethics Committee-2, August 2017. Dissemination through peer-reviewed publications and presentations.

Registration details

Trial registration number: ISRCTN15236311, date registered 09/10/2017
https://doi.org/10.1186/ISRCTN15236311

Strengths and limitations of this study:

- Prospectively designed economic evaluation of a phase III randomised controlled trial with sites across the UK
- Preference-based (utility) outcome measures at late-stage pregnancy and 6-months post-partum to enable decision-making
- 6-months post-partum follow-up is the longest we are aware of, making this a novel study

For peer review only - http://bmjopen.bmj.com/site/about/guidelines.xhtml

- Lifetime extrapolation includes costs and outcomes for both mother and infant
- Challenges relate to different smoking cessation service delivery at each trial site

INTRODUCTION

Problem

Smoking is the principal preventable cause of cancer, with 64,000 new cases annually in the UK(1). Levels of smoking reflect levels of inequality; in 2016 in England 7.9% of adults smoked in the least deprived areas compared to 27.2% in the most deprived areas(2). Lifelong smokers lose 10 years of life, however, smokers who quit before 40 years of age can expect a near to normal lifespan(3). Levels of smoking during pregnancy are high; in 2010 12% of mothers in England smoked throughout their pregnancy, this figure was 13% and 15% in Scotland and Northern Ireland respectively(4). Smoking during pregnancy is a recognised cause of low birth weight and increases the risk of premature birth by 27%(5). In addition to complications during pregnancy, after birth the consequences of continued smoking on the child are substantial: passive smoking is linked to sudden infant death syndrome, lower respiratory diseases, asthma and impaired lung function(5). Research shows that children in a house where parents or siblings smoke are 90% more likely to smoke themselves (6). Around 80% of UK women have a baby(7), most of whom are less than 40 years of age. Pregnancy is a good opportunity to help women quit smoking, and also decreases the likelihood of the baby becoming a smoker in later life(6). Helping pregnant women to quit smoking can potentially help tackle inequalities and lift families out of poverty(8). However, few of about 130,000 UK pregnant smokers quit. In the UK all pregnant women are offered NHS smoking cessation services, with free Nicotine Replacement Therapy (NRT), however, only 10% use these services and as few as 3% report abstinence at four weeks after quitting(9).

Evidence

Financial incentives are becoming a popular approach to changing behaviour that will lead to healthier lifestyles (10). A recent Cochrane review assessed incentives for smoking cessation and identified nine studies reporting results for pregnant women with a combined RR of 2.38 (95% CI 1.54 – 3.69) in favour of incentives. Eight studies were based in the US, and one in the UK. The UK study was a single site study (CPIT II) which found that offering financial incentives helped pregnant women in Glasgow, Scotland to quit(11). CPIT II estimated a cost per quality adjusted life-year of less than £500, which is considered to be highly cost-effective(12).

Aim

For peer review only - http://bmjopen.bmj.com/site/about/guidelines.xhtml

CPIT III evaluates whether the favourable findings from CPIT II can be replicated in further UK sites, and includes an additional follow-up at six-month post-partum. The CPIT III main study protocol is published elsewhere(13). The aim of the economic evaluation described here is to explore the cost-effectiveness of including financial incentives alongside usual care to increase the quit rate of pregnant women who smoke.

METHODS AND ANALYSIS

The Main RCT (CPIT III)

CPIT III is a multi-centre randomised controlled trial which aims to assess the effectiveness and cost-effectiveness of adding financial incentives to usual care to increase the smoking cessation rate among pregnant women. The primary outcome is smoking at 34-38 weeks gestation, self-reported, with those reporting as quit undertaking urine or saliva confirmatory biochemical testing. The secondary outcomes comprise: engaging with smoking cessation services and setting a quit date; smoking cessation at four weeks post quit date and six months post-partum (with those reporting as quit confirmed by biochemical testing); continuous abstinence from late pregnancy to six months post-partum; birth weight; cost-effectiveness and process evaluation.

Participants are pregnant women who self-report as smokers, are 16 years or older, less than 24 weeks pregnant and English speaking. The target recruitment number was 940, to give 90% power and 5% significance to show a doubling of quit rate from 7% in the control arm to 14% with financial incentives, allowing 15% loss to follow-up. The estimate of 7% in the control arm was derived from CPIT II (8.6% abstinent) (11, 14) and two other recent large trials of pregnancy cessation interventions, conducted in regions where CPIT III will recruit; abstinent 6.4% (15) and 7.6%(16). The average control group abstinence rate was 7.5% at late pregnancy. The estimate of 14% in the intervention arm is based on both the cessation rate in CPIT II (22.5%) plus a reflection of what is considered clinically important. A gain of 7% to 14% would be considered clinically important and is comparable to pharmaceutical aids in non-pregnant smokers (17). Assuming participants with missing smoking status are smoking as per the Russell Standard(18), the attrition rate would be 0%, and as a result 940 participants would give 94% power to detect a doubling of the quit rate from 7% to 14%.

944 participants were recruited from seven sites in England, Scotland and Northern Ireland between February 2018 and April 2020. Pregnant smokers identified at routine maternity booking appointments who were referred to smoking cessation services were assessed for eligibility. The trial was introduced to eligible women at first routine contact with smoking cessation services. Those interested in taking part were sent a participant information sheet and contacted by telephone after at least 5 days to obtain formal consent. Those consenting were randomised to either

For peer review only - http://bmjopen.bmj.com/site/about/guidelines.xhtml

receive financial incentives plus usual smoking cessation support or usual smoking cessation support only. The incentives are presented in **Error! Reference source not found.**.

Both groups will be offered local smoking cessation support and a research participation voucher. This shopping voucher for £50 is issued if data is provided for the primary outcome in late pregnancy and a further £25 shopping voucher issued if data is provided for the secondary outcome at six months post-partum. Participants can still receive the £25 shopping voucher if they provide data for the secondary outcome but do not provide data for the primary outcome.

Participants in the intervention arm will also receive up to £400 in shopping vouchers; £50 for attending an initial appointment with local smoking cessation services and setting a quit date, £50 if verified quit at 4 weeks post quit date, £100 if verified quit at 12 weeks post quit date and £200 if verified quit and provide a saliva/urine sample at 34-38 weeks gestation (primary outcome point). All incentive payments, apart from the first, require quit to be verified by carbon monoxide (CO) breath test. Cotinine/anabasine will also be used to confirm non-smoking status at the primary outcome and six months post-partum. Participants in the incentives group not attending smoking cessation services can still receive the final £200 shopping voucher if they are CO verified quit at the primary endpoint of 34-38 weeks gestation and provide a saliva/urine sample.
Further details on the CPIT III trial are detailed in the main study protocol paper which is available elsewhere (13).

Table 1: Intervention and research participation incentives

Time point	Intervention	Control
Initial local smoking cessation services meeting and setting quit date	£50 voucher	N/A
Verified quit at four weeks post quit date	£50 voucher	N/A
Verified quit at 12 weeks post quit date	£100 voucher	N/A
Verified quit at 34-38 weeks gestation and provision of urine/saliva sample if quit	£200 voucher	N/A
Providing data for primary outcome	£50 voucher for research participation	£50 voucher for research participation
Providing data for secondary outcome (quit six-month post-partum)	£25 voucher for research participation	£25 voucher for research participation

For peer review only - http://bmjopen.bmj.com/site/about/guidelines.xhtml

The Economic Evaluation

The economic evaluation will be conducted from the perspective of the UK National Health Service (NHS) and Personal Social Services (PSS), including only resources funded by the NHS, and include costs for the price year 2020.
Two analyses will be carried out; a within trial analysis and a long-term lifetime analysis. The within trial results will be presented as an incremental cost per quitter and an incremental cost per quality adjusted life-year (QALY) gained. The lifetime model results will be reported as incremental cost per quality adjusted life-year gained. For both analyses results will be assessed against the current NICE threshold of £20,000 to £30,000(19) per QALY gained. The economic evaluation will adhere to best practice and guidelines (20, 21).

An overview of this analysis is illustrated in **Error! Reference source not found.** below; the participants (pregnant smokers) are randomised to either the intervention or control arm. The primary outcome of the trial is quit at 34–38 weeks gestation and both this trial primary outcome and the economic outcome of QALYs will be used in the cost-effectiveness analysis.

Figure 1: Decision tree including intervention and outcomes

The analysis will be conducted using the statistical analysis package STATA version 16 (StataCorp. 2019. Stata Statistical Software: Release 16. College Station, TX: StataCorp LLC).

Within-trial analysis

Patient level data on resource use and outcomes will be routinely collected during the trial and used in the economic evaluation. The time horizon for the within-trial analysis is that of the trial; from time of maternity booking (less than 24 weeks gestation) to late-pregnancy (34-38 weeks gestation) and six months post-partum. No discounting of costs and outcomes is required as this time horizon is less than one year. The analysis will be undertaken based on an intention to treat (ITT) approach, and participants lost to follow up will be assumed to be smokers.

Resource use

There are four main resource use groups of relevance informing the within trial analysis: smoking cessation support (professional time), NRT, intervention costs (financial incentives) and neonatal care. Methods of data collection for these resource use categories are described in the following section.

Service delivery of smoking cessation support and prescribing of NRT is likely to vary between trial sites. Health economists will work with each site to establish the format of the service delivered at that site, and set up and maintain resource use data collection procedures. To date methods used for collecting resources include

For peer review only - http://bmjopen.bmj.com/site/about/guidelines.xhtml

For peer review only

routinely collected NHS data and bespoke Excel spreadsheets. Resource data for the cessation services will typically be around 12 weeks' worth of NRT and cessation support from first contact with cessation services to last contact.

The trial data management system will record when shopping vouchers are sent to participants and a standard postal cost will be assigned to each voucher posted. Vouchers are sent at first consultation, four and 12 weeks post-quit, late pregnancy (34-38 weeks) and six months post-partum (see Table 1). Resource use in terms of shopping vouchers will be recorded at each follow-up point, up to and including six-months post-partum.

The child's birthweight and date of delivery will be collected post-partum via the trial data management system, enabling pre-term status to be derived. Assumptions will be made about the levels of neo-natal care required based on this birthweight and prematurity/pre-term status.

Unit Costs

Unit costs will be obtained from routine sources:

- British National Formulary – NRT (22)
- Personal Social Service Research Unit – Smoking cessation advisor/pharmacist(23)
- Trial data management system – shopping vouchers/postage
- NHS reference costs, Information Services Division (ISD) NHS Scotland, and the literature – neo-natal costs.

Costs will be for the price year 2020, expressed in pounds sterling (GBP £) and inflated if necessary using the Health Services (HS) index(24). For publication in journals, final costs may also be presented in US dollars, converted using the exchange rate, so that the results will be appropriate for an international audience.

Unit cost information will be combined with the resource use data collected in the trial to estimate the total cost per participant in each trial arm. These participant total costs will be aggregated to estimate the total cost of each trial arm and subsequently the average cost per participant for each intervention.

Regression analysis will be conducted to explore the effect that baseline variables have on the cost of each intervention, such as EQ-5D-5L score, site, gestational age at booking. Variables included will be in line with the statistical analysis plan and main analysis.

Outcomes

There will be two economic outcomes: the within trial quit rate and QALY.

The primary outcome of the trial is smoking status at 34-38 weeks gestation. This indicator will inform the incremental cost per quitter analysis. As this is an

For peer review only - http://bmjopen.bmj.com/site/about/guidelines.xhtml

intermediate outcome and not a measure of health we are also conducting a within trial incremental cost per QALY analysis.

The QALY is a combination of quality of life and quantity of life. Quality of life will be measured using the EQ-5D-5L questionnaire (25), which measures five dimensions: mobility, self-care, usual activities, pain/discomfort and anxiety/depression, with five levels: no problems, slight problems, moderate problems, severe problems and extreme problems. The EQ-5D-5L will be completed by participants at three time points: baseline, late pregnancy (34-38 weeks gestation) and six months post-partum.

The EQ-5D-5L is converted to utilities using value sets giving a utility value of between -0.59 and 1. A utility value of 1 represents full health, 0 represents death and between 0 and -0.59 represents a state worse than death (where members of the general public were asked to value health states using time trade off, one option was believed to be worse than death, i.e. bowel and bladder incontinence). We will map the EQ-5D-5L results to the EQ-5D-3L UK value set (26) in accordance to the recommendation in the current position statement from NICE (27, 28).
The quantity of life element of the QALY will be the length of time the participant remains in the trial, using standard QALY calculation applying an area under the curve approach(29). For example, for a participant recruited at 20 weeks gestation who has primary outcome data collection at 38 weeks the calculation for a quality adjusted life-year would be as follows:

$$(\text{Utility at 38 weeks gestation - Utility at 20 weeks gestation}) \times \frac{38-20}{52}$$

When combining the utility score generated from the EQ-5D-5L with quantity of life to give a QALY, standard area-under-the-curve methods(30) will be employed; weighting utilities by the duration of each time interval. Any change in utility between time points will be treated as linear.

A regression analysis will be carried out in line with the main analysis and statistical analysis plan exploring the effect that baseline variables have on the outcomes, such as site, gestational age at booking and in particular baseline utilities(29).

Analysis of costs and effects

Missing health economic data will be dealt with in line with best practice guidance(31): a descriptive analysis of the missing data will be carried out and assumptions will be made to ascertain the type of missingness, following this an appropriate method for handling missing data will be chosen. If data is missing at random we will use multiple imputation using chained equations. Finally, a sensitivity analysis for complete cases will be conducted and further sensitivity analyses will be carried out around assumptions as appropriate, for example using best/worst case assumption scenarios and exploring unexpected issues arising such

For peer review only - http://bmjopen.bmj.com/site/about/guidelines.xhtml

as COVID-19 and not being able to take CO monitoring readings . Any participants with missing outcome data will be assumed to be smokers.

The cost-effectiveness analysis will bring together the estimates of cost and effect as described above, by estimating an incremental cost effectiveness ratio (ICER) of cost per quitter and per QALY. The difference between mean costs will be divided by the difference in mean QALYs and quit rates to calculate the ICER, the formula for this is presented below. The control arm is denoted by 'A' and intervention (financial incentives) arm by 'B'.

$$ICER = \frac{C_B - C_A}{E_B - E_A}$$

The costs, QALYs and quit rates per participant in each trial arm (i.e. the average quit rate per arm) will be presented as means and standard deviations. Differences in means between trial arms will be presented with 95% confidence intervals (CI) using generalised linear models with appropriate link and family functions.

Uncertainty

The effect of uncertainty on the results will be explored using non-parametric bootstrapping techniques and the resulting samples will be plotted on a cost-effectiveness plane in order to graphically represent uncertainty (32). Cost-effectiveness acceptability curves (CEACs) will then be used to present uncertainty in terms of willingness to pay under various monetary thresholds (33). 95% confidence intervals for the ICER will be presented using the results of the bootstrapping. If appropriate, 95% confidence intervals for the ICER will be estimated using Fieller's Theorem, a technique that includes any correlation between cost and outcome(34).

Any sub-group analyses deemed appropriate in the statistical analysis plan will also be considered for the economic analysis. This will include an analysis using self-reported smoker/quitter extracted from case notes where the trial team have not been able to establish smoking status via direct contact with participants.

Two sensitivity analyses will be run; using miscarriage data from the trial and exploring the effects of gaming (where the participant self-reports as a quitter but is still smoking) in the trial.

Long-term model

The time horizon for the long-term economic model is lifetime. Costs and effects will be discounted at 3.5% annually in line with recommendations from NICE(21).
The within trial analysis only considers the costs and effects of the pregnant woman and neonatal costs. The long-term model will also include the costs and effects related to the child. The rationale for this is that children of smokers are more likely to smoke themselves and will also be exposed to second-hand smoke, increasing the likelihood of smoking related diseases(6).

For peer review only - http://bmjopen.bmj.com/site/about/guidelines.xhtml

For peer review only

Model structure

The analysis will utilise and adapt a published probabilistic decision analytic Markov model, the Economics of Smoking in Pregnancy (ESIP) model (35). The model will comprise two sections; one to capture the lifetime costs and outcomes for the mother, and one to capture the lifetime costs and outcomes of the child(21). The ESIP model was the first model specifically developed to include future potential cost savings and improvement in health for both mother and child. A short-term cost-effectiveness analysis can be misleading as the benefits of stopping smoking are not captured in a short time horizon, which is why it is important to incorporate future benefits to assess the full impact of offering financial incentives to pregnant smokers.

The mother's portion of the model will include: the costs of smoking cessation services, financial incentives, NRT use and smoking related diseases (coronary heart disease, COPD, lung cancer and stroke that account for 75% of smoking related deaths), plus data on relapse rates and transition to smoking related diseases if the woman relapses. The child's part of the model will include: costs associated at birth for children with smoking mothers if appropriate (premature birth and underweight new-borns), outcomes related to exposure to second-hand smoke in the home (i.e. asthma and longer term smoking related diseases), smoking uptake rates for children of smokers and associated smoking related diseases.

The trial quit rate at six months post birth will be used to predict smoking relapse post-partum. The final utility estimates for patient level data from the post-partum follow-up will also be used as a point for extrapolation for lifetime analysis (rather than assuming background population utility values).

Figure 2 depicts the likely structure of the long-term model(36). The model is split between mother and infant; the first 'Within pregnancy' section on the left illustrates within pregnancy outcomes and related costs, the middle 'Childhood' section depicts the infant's childhood from birth to 15 years old and the last section 'Lifetime' on the right, depicts lifetime for both mother and infant.

Resource use and outcomes collected during the trial will be extrapolated and supplemented with published costs from the literature.

Figure 2: Model structure (Jones et al) (36)

Analysis

The lifetime analysis results will be presented as cost per QALY gained for both the mother and child, separately and combined.

A probabilistic sensitivity analysis will be included and appropriate sensitivity analyses will explore underlying assumptions used in the model, including:

- Re-analysis to account for gaming identified
- Self-reported outcomes
- Varying amount of incentive

For peer review only - http://bmjopen.bmj.com/site/about/guidelines.xhtml

- Applying alternative discount rates
- Varying relapse rates

Patient and Public Involvement

No patient involved.

Ethics and dissemination

Ethics approval was received from NHS West of Scotland Research Ethics Committee 15th August 2017. Results of the main trial and economic evaluation will be disseminated through peer-reviewed publications and presentations.

References

1. Parkin DM, Boyd L, Walker LC. The fraction of cancer attributable to lifestyle and environmental factors in the UK in 2010 Summary and conclusions. British Journal of Cancer. 2011;105:S77-S81.
2. Office of National Statistics. Likelihood of smoking four times higher in Englandâ€™s most deprived areas than least deprived - Office for National Statistics 2019 [Available from: https://www.ons.gov.uk/peoplepopulationandcommunity/healthandsocialcare/drugusealcoholandsmoking/articles/likelihoodofsmokingfourtimeshigherinenglandsmostdeprivedareasthanleastdeprived/2018-03-14.
3. Doll R, Peto R, Boreham J, Sutherland I. Mortality in relation to smoking: 50 years' observations on male British doctors. Bmj-British Medical Journal. 2004;328(7455):1519-28.
4. HSCIC Health and Social Care Information Centre. Infant feeding survey 2010 2011 [Available from: https://digital.nhs.uk/data-and-information/publications/statistical/infant-feeding-survey/infant-feeding-survey-2010-early-results.
5. Physicians RCo. Passive smoking and children. A report by the Tobacco Advisory Group London2010 [Available from: https://www.rcplondon.ac.uk/news/passive-smoking-major-health-hazard-children-says-rcp.
6. Leonardi-Bee J, Jere ML, Britton J. Exposure to parental and sibling smoking and the risk of smoking uptake in childhood and adolescence: a systematic review and meta-analysis. Thorax. 2011;66(10):847-55.
7. Portani M, Whitworth S. A comparison of the characteristics of childless women and mother in the ONS longitudinal study. Population trends. 2009(136):10-20.
8. Belvin C, Britton J, Holmes J, Langley T. Parental smoking and child poverty in the UK: an analysis of national survey data. Bmc Public Health. 2015;15.
9. Tappin DM, MacAskill S, Bauld L, Eadie D, Shipton D, Galbraith L. Smoking prevalence and smoking cessation services for pregnant women in Scotland. Substance Abuse Treatment Prevention and Policy. 2010;5.
10. Vlaev I, King D, Darzi A, Dolan P. Changing health behaviors using financial incentives: a review from behavioral economics. Bmc Public Health. 2019;19(1).
11. Tappin D, Bauld L, Purves D, Boyd K, Sinclair L, MacAskill S, et al. Financial incentives for smoking cessation in pregnancy: randomised controlled trial. BMJ-British Medical Journal. 2015;350.
12. Boyd KA, Briggs AH, Bauld L, Sinclair L, Tappin D. Are financial incentives cost-effective to support smoking cessation during pregnancy? Addiction. 2016;111(2):360-70.

For peer review only - http://bmjopen.bmj.com/site/about/guidelines.xhtml

13. Sinclair L, McFadden M, Tilbrook H, Mitchell A, Keding A, Watson J, et al. The smoking cessation in pregnancy incentives trial (CPIT): study protocol for a phase III randomised controlled trial. Trials. 2020;21(1).
14. Tappin D, Bauld L, Purves D, Boyd K, Sinclair L, MacAskill S, et al. Financial incentives for smoking cessation in pregnancy: Randomised controlled trial. BMJ (Online). 2015;350 (no pagination)(h134).
15. Ussher M, Lewis S, Aveyard P, Manyonda I, West R, Lewis B, et al. Physical activity for smoking cessation in pregnancy: randomised controlled trial. Bmj-British Medical Journal. 2015;350.
16. Coleman T, Cooper S, Thornton JG, Grainge MJ, Watts K, Britton J, et al. A Randomized Trial of Nicotine-Replacement Therapy Patches in Pregnancy. New England Journal of Medicine. 2012;366(9):808-18.
17. Cahill K, Stevens S, Perera R, Lancaster T. Pharmacological interventions for smoking cessation: an overview and network meta-analysis. The Cochrane database of systematic reviews. 2013(5):CD009329-CD.
18. West R, Hajek P, Stead L, Stapleton J. Outcome criteria in smoking cessation trials: proposal for a common standard. Addiction. 2005;100(3):299-303.
19. National Institute for Health and Care Excellence. Interim methods guide for developing service guidance 2014: NICE; 2016 [Available from: https://www.nice.org.uk/process/pmg8/chapter/5-developing-a-conceptual-model.
20. Husereau D, Drummond M, Petrou S, Carswell C, Moher D, Greenberg D, et al. Consolidated Health Economic Evaluation Reporting Standards (CHEERS) statement. European Journal of Health Economics. 2013;14(3):367-72.
21. Excellence NIfHaC. Guide to the methods of technology appraisal 2013 | foreword | Guidance and guidelines | NICE. 2015.
22. Joint Formulary Committee. British National Formulary 2018 [Available from: https://www.medicinescomplete.com/#/.
23. Curtis L, Burns A. Unit Costs of Health and Social Care 2017 | PSSRU 2018 [Available from: https://www.pssru.ac.uk/project-pages/unit-costs/unit-costs-2017/.
24. Curtis L. Unit Costs of Health and Social Care 2018 | PSSRU 2019 [Available from: https://www.pssru.ac.uk/project-pages/unit-costs/unit-costs-2018/.
25. Herdman M, Gudex C, Lloyd A, Janssen MF, Kind P, Parkin D, et al. Development and preliminary testing of the new five-level version of EQ-5D (EQ-5D-5L). Quality of Life Research. 2011;20(10):1727-36.
26. van Hout B, Janssen MF, Feng Y-S, Kohlmann T, Busschbach J, Golicki D, et al. Interim Scoring for the EQ-5D-5L: Mapping the EQ-5D-5L to EQ-5D-3L Value Sets. Value in Health. 2012;15(5):708-15.
27. National Institute for Health and Care Excellence. Position statement on use of the EQ-5D-5L valuation set for England (updated November 2018) | NICE technology appraisal guidance | NICE guidance | Our programmes | What we do | About | NICE [CorporatePage]. NICE; 2018 [updated 2018-11-29. Available from: https://www.nice.org.uk/about/what-we-do/our-programmes/nice-guidance/technology-appraisal-guidance/eq-5d-5l.
28. Hernandez-Alava M, Pudney S. eq5dmap: A command for mapping between EQ-5D-3L and EQ-5D-5L. Stata Journal. 2018;18(2):395-415.
29. Manca A, Hawkins N, Sculpher MJ. Estimating mean QALYs in trial-based cost-effectiveness analysis: the importance of controlling for baseline utility. Health Economics. 2005;14(5):487-96.
30. Billingham LJ, Abrams KR, Jones DR. Methods for the analysis of quality-of-life and survival data in health technology assessment. Health technology assessment (Winchester, England). 1999;3(10):1-152.
31. Faria R, Gomes M, Epstein D, White IR. A Guide to Handling Missing Data in Cost-Effectiveness Analysis Conducted Within Randomised Controlled Trials. Pharmacoeconomics. 2014;32(12):1157-70.

For peer review only - http://bmjopen.bmj.com/site/about/guidelines.xhtml

32. Briggs A, Claxton K, Sculpher M. Decision Modelling for Health Economic Evaluation. 2011 ed. Gray A, Briggs A, editors. New York: Oxford University Press; 2006.
33. Fenwick E, Claxton K, Sculpher M. Representing uncertainty: The role of cost-effectiveness acceptability curves. Health Economics. 2001;10(8):779-87.
34. Fieller EC. SOME PROBLEMS IN INTERVAL ESTIMATION. Journal of the Royal Statistical Society Series B-Statistical Methodology. 1954;16(2):175-85.
35. Jones M, Smith M, Lewis S, Parrott S, Coleman T. A dynamic, modifiable model for estimating cost-effectiveness of smoking cessation interventions in pregnancy: application to an RCT of self-help delivered by text message. Addiction. 2019;114(2):353-65.
36. Jones Mea. Economics of Smoking in Pregnancy (ESIP) Model - The University of Nottingham 2019 [Available from: https://www.nottingham.ac.uk/research/groups/tobaccoandalcohol/smoking-in-pregnancy/esip/index.aspx.

Trial registration

Current Controlled Trials ISRCTN15236311 date registered 09/10/2017
https://doi.org/10.1186/ISRCTN15236311

Authors' contributions

NM and KAB designed the economic evaluation protocol and wrote the paper, LS, LB, DT and AM contributed to revising the content of the paper and contributed to editing.

Source of funding

Cancer research UK (C48006/A20863), Chief Scientist Office Scottish Government (HIPS/16/1), Health and Social Care Services Northern Ireland (COM/5352/17), Chest Heart & Stroke Northern Ireland (2019_09), Lullaby Trust (272), Scottish Cot Death Trust (no reference available), Public Health Agency NI (no reference available).

Competing interests statement

No authors have competing interests.

For peer review only - http://bmjopen.bmj.com/site/about/guidelines.xhtml

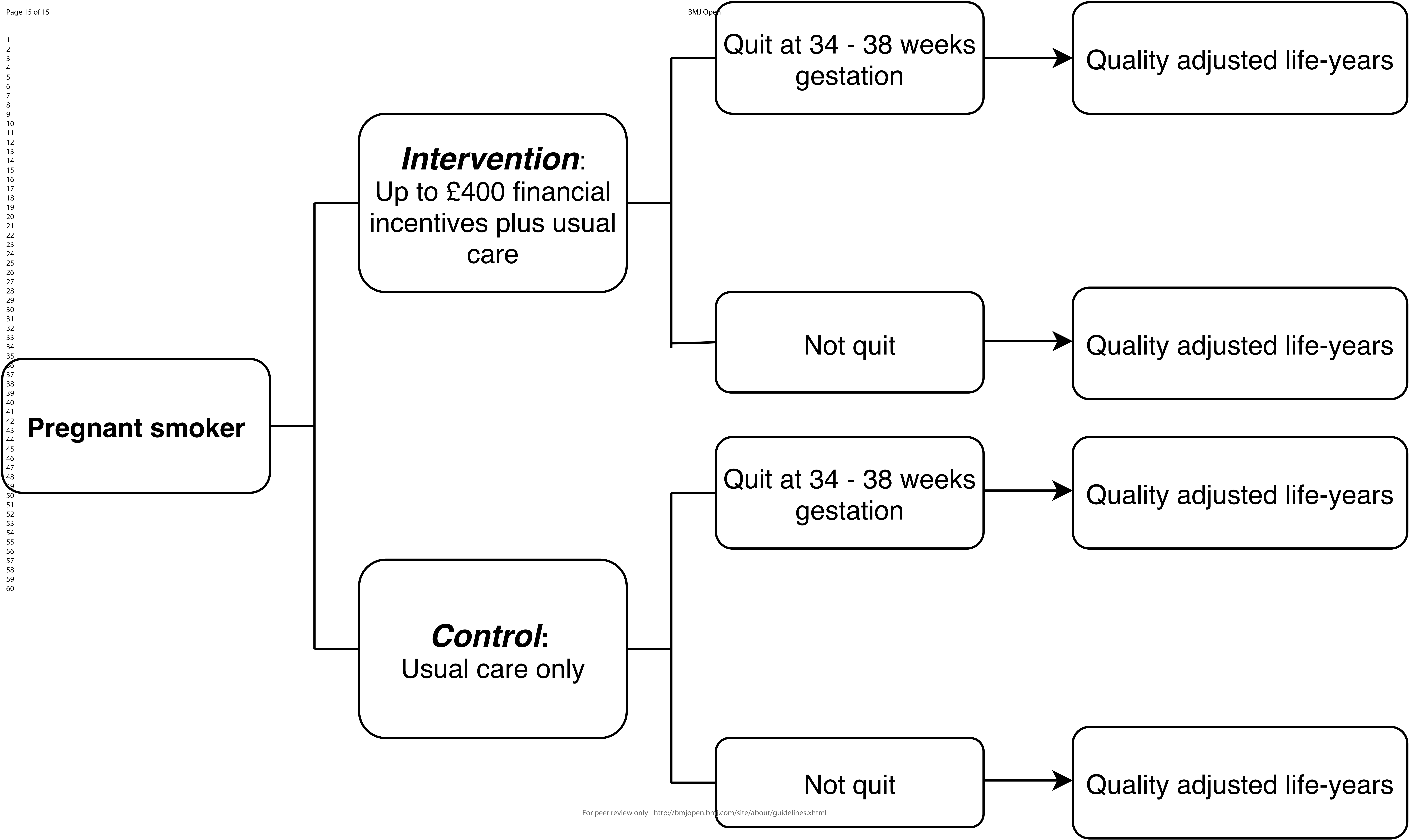
Pregnant smoker
Intervention: Up to £400 financial incentives plus usual care
Control: Usual care only
Quit at 34 - 38 weeks gestation
Not quit
Quit at 34 - 38 weeks gestation
Not quit
Quality adjusted life-years
Quality adjusted life-years
Quality adjusted life-years
Quality adjusted life-years

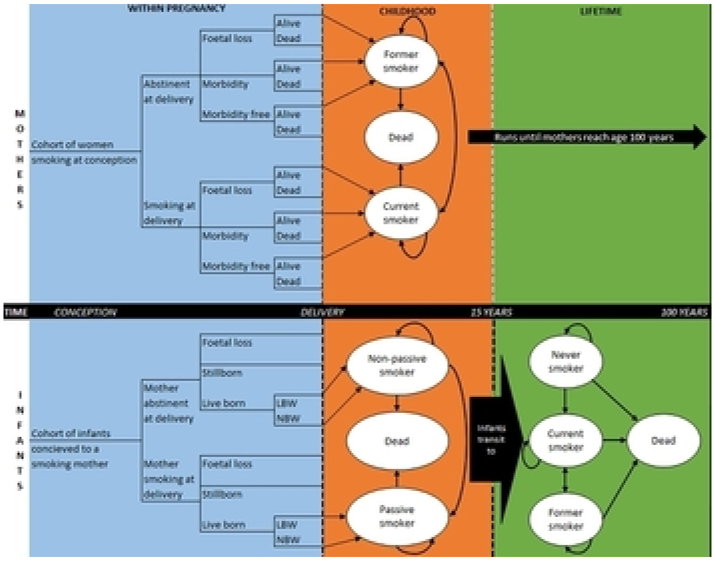

Figure 2 Model structure

14x11mm (600 x 600 DPI)
